# Supplementary material for: Suicide on YouTube:Factors engaging viewers to a selection of suicide-themed videos
Source: PLoS One. 2021 Jun 10;16(6):e0252796. doi: 10.1371/journal.pone.0252796 (PMC8191908; doi:10.1371/journal.pone.0252796)
Supplement: S1 Codebook — (DOCX) [file pone.0252796.s001.docx]

**Codebook**

**Identification**

**I1. Video ID** : Each video is given a number between 1 to 100

- I1-1. URL
- I1-2. Title
- I1-3. Description
- I1-4. Upload Date

**Factor 1: Who (Characteristics of deliverer)**

**F1. Creator** (who uploaded the video or who owns the channel)

| 1 | Suicide-prevention organization |
| --- | --- |
| 2 | Clinic and health organization |
| 3 | News agency |
| 4 | One-person creator |
| 5 | Production organization (music/film/documentaries) |
| 6 | Educational facilities |
| 7 | Religious group |
| 8 | Others (memo: write in words or sentences) |

**F2. Message Deliverer** (who is the person talking about suicide)

| 1 | Survivors |
| --- | --- |
| 2 | Family member who lost the beloved ones to suicide |
| 3 | Friends who lost the beloved ones to suicide |
| 4 | News personnel |
| 5 | Rescuer |
| 6 | Narrator |
| 7 | Artist-musician, film personnel, dancer |
| 8 | Lecturer/Educator |
| 9 | Medical personnel |
| 10 | One-person creator |
| 11 | Others (memo: write in words or sentences) |

**F3. Anonymity**

| 1 | Real name provided |
| --- | --- |
| 2 | Anonymous |

**F4. Deliverer Demographics (F4-1 / F4-2 / F4-3)**

- **F4-1. Nationality of the deliverer**

| 1 | Unknown |
| --- | --- |
| 2 | American |
| 3 | Korean |
| 4 | Japanese |
| 5 | Indian |
| 6 | Zagreb / Croatia |
| 7 | Australia |
| 8 | UK / England |
| 9 | Canadian |
| 10 | Africa / Kenya |

- **F4-2. Gender of the deliverer**

| 1 | Male |
| --- | --- |
| 2 | Female |
| 3 | Multiple people: mix |
| 4 | Others (No human being in the video) |

- **F4-3. Age of the deliverer**

| 1 | 0-10 |
| --- | --- |
| 2 | 11-20 |
| 3 | 21-30 |
| 4 | 31-40 |
| 5 | 41-50 |
| 6 | 51-60 |
| 7 | 61 and above |
| 8 | Unknown |

**Factor 2: What (Characteristics of stories-Content)**

**F5. Public Figure** (whether the message is about suicide stories of celebrities or famous people (Phillips, 1974; Stack, 2000))

| 1 | Celebrity |
| --- | --- |
| 2 | Politician |
| 3 | YouTuber |
| 4 | Non-celebrity |
| 5 | Others (Not about an actual suicide story) |

**F6. Real vs. Fictional** (whether the content is about a real suicide story (Stack, 2000))

| 1 | Real |
| --- | --- |
| 2 | Fictional |
| 3 | Unidentifiable: Not about specific suicide incident, but about general suicide |

**F7. Suicide Attempt vs. Complete Suicide** (Stack, 2000)

| 1 | Suicide attempt |
| --- | --- |
| 2 | Complete suicide |
| 3 | Suicide ideation |

**Factor 3: How (Characteristics of stories-Expression)**

**F8. General information**

- **F8-1. Language**

| 1 | English |
| --- | --- |
| 2 | Korean |
| 3 | Japanese |
| 4 | French |
| 5 | Indian |
| 6 | No language |

- **F8-2. Length of Video (n’N’’)**
- **F8-3. Advertisement**

| 1 | Yes |
| --- | --- |
| 2 | No |

**F9. Expression Guidelines**

| 1 | Graphic expression or illustration of how-to |
| --- | --- |
| 2 | Verbal expression |
| 3 | Textual expression |
| 4 | None |
| 5 | Multiple expressions (memo: specify which ones) |

**F10-1. Existence of Warning Sign**

| 1 | O |
| --- | --- |
| 2 | X |
| 3 | YouTube platform’s warning sign: “The following content has been identified by the YouTube community as inappropriate or offensive to some audiences. Viewer discretion is advised.”  Viewers can watch the content after pressing the button ‘I UNDERSTAND AND WITH TO PROCEED’ |

**F10-2. Placement of Warning Sign**

| 1 | In the title |
| --- | --- |
| 2 | In the thumbnail |
| 3 | In description |
| 4 | In the first-half of the video |
| 5 | In the other half of the video |
| 6 | None |

**F11-1. Existence of Hotline**

| 1 | O |
| --- | --- |
| 2 | X |

**F11-2. Placement of Hotline**

| 1 | In the title |
| --- | --- |
| 2 | In the thumbnail |
| 3 | In description |
| 4 | In the first-half of the video |
| 5 | In the other half of the video |
| 6 | None |
| 7 | Multiple (memo: specify which ones) |

**F12. Genre**

| 1 | Entertainment |
| --- | --- |
| 2 | People & Blog |
| 3 | News & Politics |
| 4 | Music |
| 5 | Science & Technology |
| 6 | Film & Animation |
| 7 | Gaming |
| 8 | Nonprofits & Activism |
| 9 | Education |
| 10 | Others |

**Dependent Variables: Popularity Metrics**

**D1. Number of Video Views**

**D2. Number of Comments**

**D3. Ratings**

**D4. Number of Likes**

**D5. Number of Dislikes**

**D6. Average Ratings**
