# Supplementary material for: Suicide on YouTube:Factors engaging viewers to a selection of suicide-themed videos
Source: PLoS One. 2021 Jun 10;16(6):e0252796. doi: 10.1371/journal.pone.0252796 (PMC8191908; doi:10.1371/journal.pone.0252796)
Supplement: S2 File — (DOCX) [file pone.0252796.s003.docx]

**Codebook**

| **Video ID**: **Each video is given a number between 1 to 100** | | |
| --- | --- | --- |
| **Factor 1: Who (Characteristics of deliverer)** | | |
| **F1. Creator** | 1 | Suicide-prevention organization |
|  | 2 | Clinic and health organization |
|  | 3 | News agency |
|  | 4 | One-person creator |
|  | 5 | Production organization (music/film/documentaries) |
|  | 6 | Educational facilities |
|  | 7 | Religious group |
|  | 8 | Others |
| **F2. Message Deliverer** | 1 | Survivors |
|  | 2 | Family member who lost the beloved ones to suicide |
|  | 3 | Friends who lost the beloved ones to suicide |
|  | 4 | News personnel |
|  | 5 | Rescuer |
|  | 6 | Narrator |
|  | 7 | Artist-musician, film personnel, dancer |
|  | 8 | Lecturer/Educator |
|  | 9 | Medical personnel |
|  | 10 | One-person creator |
|  | 11 | Others |
| **F3. Anonymity** | 1 | Real name provided |
|  | 2 | Anonymous |
| **Factor 2: What (Characteristics of stories-Content)** | | |
| **F4. Number of stories** | | |
| **F5. Public Figure** | 1 | Celebrity |
|  | 2 | Politician |
|  | 3 | YouTuber |
|  | 4 | Non-celebrity |
|  | 5 | Others |
| **F6. Real vs. Fictional** | 1 | Real |
|  | 2 | Fictional |
|  | 3 | Unidentifiable: Not about specific suicide incident, but about general suicide |
| **F7. Type of Suicide** | 1 | Suicide attempt |
|  | 2 | Complete suicide |
|  | 3 | Suicide ideation |
| **Factor 3: How (Characteristics of stories-Expression)** | | |
| **F8. Advertisement** | 1 | Yes |
|  | 2 | No |
| **F9. Expression Guidelines** | 1 | Graphic expression or illustration of how-to |
|  | 2 | Verbal expression |
|  | 3 | Textual expression |
|  | 4 | None |
|  | 5 | Multiple expressions (memo: specify which ones) |
| **F10-1. Existence of Warning Sign** | 1 | O |
|  | 2 | X |
|  | 3 | YouTube platform’s warning sign: “The following content has been identified by the YouTube community as inappropriate or offensive to some audiences. Viewer discretion is advised.”  Viewers can watch the content after pressing the button ‘I UNDERSTAND AND WITH TO PROCEED’ |
| **F10-2. Placement of Warning Sign** | 1 | In the title |
|  | 2 | In the thumbnail |
|  | 3 | In description |
|  | 4 | In the first-half of the video |
|  | 5 | In the other half of the video |
|  | 6 | None |
| **F11-1. Existence of Hotline** | 1 | O |
|  | 2 | X |
| **F11-2. Placement of Hotline** | 1 | In the title |
|  | 2 | In the thumbnail |
|  | 3 | In description |
|  | 4 | In the first-half of the video |
|  | 5 | In the other half of the video |
|  | 6 | None |
|  | 7 | Multiple (memo: specify which ones) |
| **F12. Genre** | 1 | Entertainment |
|  | 2 | People & Blog |
|  | 3 | News & Politics |
|  | 4 | Music |
|  | 5 | Science & Technology |
|  | 6 | Film & Animation |
|  | 7 | Gaming |
|  | 8 | Nonprofits & Activism |
|  | 9 | Education |
|  | 10 | Others |
